# The phosphorylated prodrug FTY720 is a histone deacetylase inhibitor that reactivates ERα expression and enhances hormonal therapy for breast cancer

**DOI:** 10.1038/oncsis.2015.16

**Published:** 2015-06-08

**Authors:** N C Hait, D Avni, A Yamada, M Nagahashi, T Aoyagi, H Aoki, C I Dumur, Z Zelenko, E J Gallagher, D Leroith, S Milstien, K Takabe, S Spiegel

**Affiliations:** 1 Department of Biochemistry and Molecular Biology, Virginia Commonwealth University School of Medicine, Richmond, VA, USA; 2 Department of Surgery, Division of Surgical Oncology, Virginia Commonwealth University School of Medicine, Richmond, VA, USA; 3 Department of Pathology, and Massey Cancer Center, Virginia Commonwealth University School of Medicine, Richmond, VA, USA; 4 Division of Endocrinology, Diabetes and Bone Diseases, Icahn School of Medicine at Mt Sinai, New York, NY, USA

## Abstract

Estrogen receptor-α (ERα)-negative breast cancer is clinically aggressive and does not respond to conventional hormonal therapies. Strategies that lead to re-expression of ERα could sensitize ERα-negative breast cancers to selective ER modulators. FTY720 (fingolimod, Gilenya), a sphingosine analog, is the Food and Drug Administration (FDA)-approved prodrug for treatment of multiple sclerosis that also has anticancer actions that are not yet well understood. We found that FTY720 is phosphorylated in breast cancer cells by nuclear sphingosine kinase 2 and accumulates there. Nuclear FTY720-P is a potent inhibitor of class I histone deacetylases (HDACs) that enhances histone acetylations and regulates expression of a restricted set of genes independently of its known effects on canonical signaling through sphingosine-1-phosphate receptors. High-fat diet (HFD) and obesity, which is now endemic, increase breast cancer risk and have been associated with worse prognosis. HFD accelerated the onset of tumors with more advanced lesions and increased triple-negative spontaneous breast tumors and HDAC activity in MMTV-PyMT transgenic mice. Oral administration of clinically relevant doses of FTY720 suppressed development, progression and aggressiveness of spontaneous breast tumors in these mice, reduced HDAC activity and strikingly reversed HFD-induced loss of estrogen and progesterone receptors in advanced carcinoma. In ERα-negative human and murine breast cancer cells, FTY720 reactivated expression of silenced ERα and sensitized them to tamoxifen. Moreover, treatment with FTY720 also re-expressed ERα and increased therapeutic sensitivity of ERα-negative syngeneic breast tumors to tamoxifen *in vivo* more potently than a known HDAC inhibitor. Our work suggests that a multipronged attack with FTY720 is a novel combination approach for effective treatment of both conventional hormonal therapy-resistant breast cancer and triple-negative breast cancer.

## Introduction

The majority of breast tumors express the estrogen receptor-α (ERα) that plays important roles in breast cancer pathogenesis and progression, and hormonal therapies such as tamoxifen (TAM) are the first line of adjuvant therapy.^1, 2^ Unfortunately, 30% of these patients will ultimately fail therapy because of *de novo* or acquired resistance. Moreover, patients with ER, progesterone receptor (PR) and human epidermal growth factor receptor 2 (also known as ErbB-2) triple-negative breast cancer, which is aggressive with high recurrence, metastatic and mortality rates,^3^ do not respond to hormonal therapies and have limited treatment options. Epidemiological and clinical studies indicate that obesity, which is now endemic, increases breast cancer risk and is associated with worse prognosis^4^ that may be due in part to the high frequency of triple-negative breast cancer and ineffectual hormonal therapy.^5^ As hormonal therapy is so effective with relatively few side effects, the possibility of reversing hormonal unresponsiveness is an appealing treatment approach. Histone deacetylases (HDACs) are negative regulators of ERα transcription, and HDAC inhibitors, including suberoylanilide hydroxamic acid (SAHA, vorinostat) and trichostatin A, have been shown to reactivate ERα expression in ERα−negative breast cancer cells and reverse TAM resistance in preclinical studies.^6, 7, 8^ Encouragingly, in phase II clinical trials, the combination of vorinostat and TAM showed promising activity in reversing hormone resistance.^9^

FTY720 (fingolimod), the Food and Drug Administration (FDA) approved prodrug for the treatment of multiple sclerosis, is phosphorylated *in vivo* by sphingosine kinase 2 (SphK2) to its active form FTY720-phosphate (FTY720-P), a mimetic of sphingosine-1-phosphate (S1P) and an agonist of four S1P receptors (S1PRs) that interferes with immune cell trafficking by inducing internalization and degradation of S1PR1.^10^ However, FTY720 has strong anticancer effects *in vitro* and *in vivo* in various types of cancers including breast^11, 12^ that are not well understood independently of its effects on immune cell trafficking.^13^ Although some of its actions have been attributed to FTY720-P acting as a functional antagonist of S1PR1, reducing persistent activation of the transcription factor STAT3 (signal transducer and activator of transcription 3) important in malignant progression,^14, 15, 16^ others have shown that the unphosphorylated FTY720 is an activator of protein phosphatase 2A, a tumor suppressor that is inactivated in many cancers.^17, 18^ However, our recent study suggests that FTY720-P and not FTY720 binds and inhibits recombinant class I HDACs.^19^ Because it is generally believed that FTY720 in cancer cells is phosphorylated at the plasma membrane by SphK2 to form FTY720-P that acts via S1PRs, we asked where FTY720 is phosphorylated in breast cancer cells and whether FTY720-P also inhibits HDACs in these cells and in tumors to regulate histone acetylation and gene expression, and can be used to re-express ERα in ER-negative aggressive breast carcinoma for hormonal therapies.

## Results

### SphK2 produces FTY720-P in the nucleus of breast cancer cells that inhibits class I HDACs

**Figure 1:**
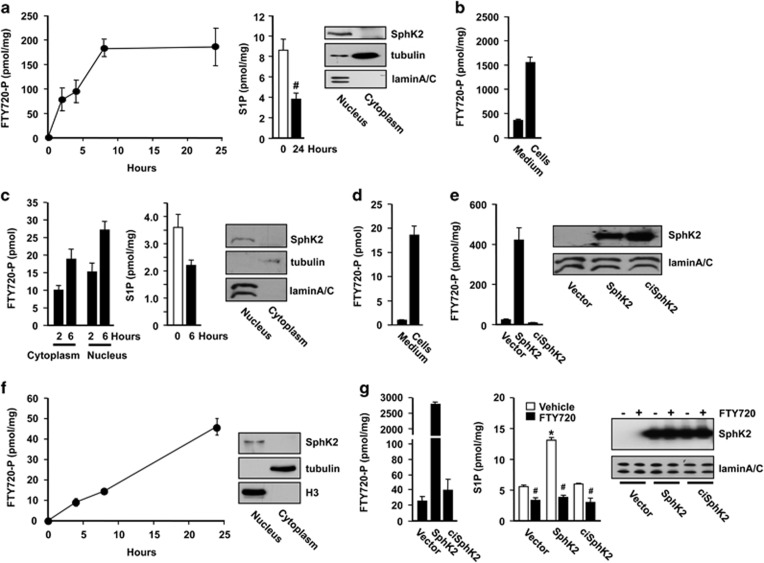
FTY720-P is produced in the nucleus of breast cancer cells by SphK2. Breast cancer cell lines, murine 4T1 (**a, b**), human MDA-MB-231 (**c, d**) and human MCF7 (**f**) were treated with 5 μM FTY720. (**a, c**) Nuclear levels of FTY720-P and S1P were determined by liquid chromatography, electrospray ionization/tandem mass spectrometry (LC-ESI-MS/MS) at the indicated times. Equal amounts of protein from nuclear and cytosolic fractions were analyzed by immunoblotting with SphK2 antibody. Antibodies against histone H3 or laminA/C and tubulin were used as nuclear and cytosol markers. (**b**, **d**) Total intracellular and secreted FTY720-P were determined in 4T1 cells after 8 h and MDA-MB-231 cells after 6 h of FTY720 treatment, respectively. MDA-MB-231 cells (**e**) and MCF7 cells (**g**) transfected with vector, SphK2 or catalytically inactive SphK2^G212E^ (ciSphK2) were treated with vehicle or 5 μM FTY720 for 6 and 24 h, respectively. Nuclear levels of FTY720-P and S1P were determined by LC-ESI-MS/MS. Data are mean±s.d. *P<0.005 compared with vector; ^#^*P*<0.005 compared with vehicle. Equal expression of nuclear SphK2 was confirmed by immunoblotting.

Following treatment with FTY720, an analog of sphingosine, FTY720-P is produced and accumulates over time in the nucleus of human and murine breast cancer cells in agreement with the predominant nuclear localization of SphK2 in these cells (Figures 1a, c, and f). Nuclear S1P levels were concomitantly decreased by almost twofold in these cell lines after FTY720 treatment because of decreased phosphorylation of the endogenous substrate sphingosine (Figures 1a and c). Interestingly, although it is generally assumed that most of the actions of the phosphorylated active form of FTY720 are at the plasma membrane to modulate S1PR signaling,^10^ much more FTY720-P was present in cells than secreted into the media where it can interact with S1PRs (Figures 1b and d). Overexpression of SphK2 robustly increased the formation of nuclear FTY720-P by >20-fold in MDA-MB-231 cells (Figure 1e) and 100-fold in MCF7 cells (Figure 1g), whereas catalytically inactive SphK2^G212E^ had no significant effect on phosphorylation of FTY720 or formation of nuclear S1P. In agreement with our previous results showing that FTY720-P inhibits recombinant class I HDACs,^19^ we observed that FTY720-P but not FTY720 itself inhibited endogenous class I HDACs (HDAC1–3 and HDAC8) immunoprecipitated from nuclear extracts with the corresponding antibodies as potently as the pan HDAC inhibitor SAHA (Supplementary Figure S1). However, in contrast to SAHA that inhibited class II HDAC7, FTY720-P did not.

### Phosphorylated FTY720 enhances histone acetylations and regulates gene expression independently of S1PRs

**Figure 2:**
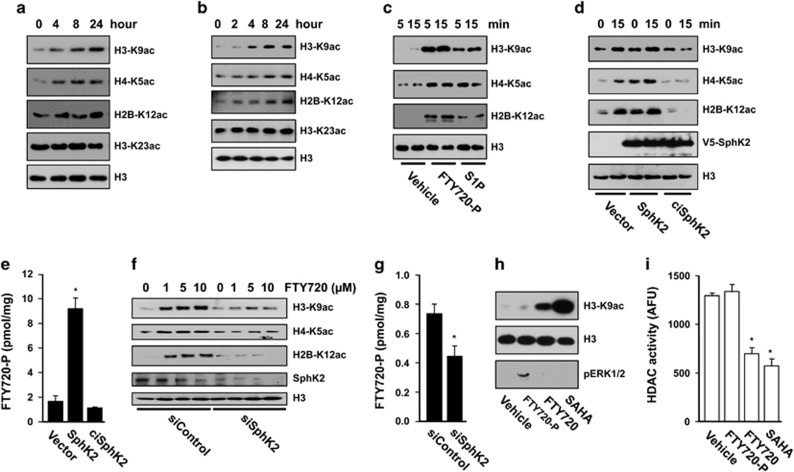
Nuclear FTY720-P enhances specific histone acetylations in breast cancer cells. MCF7 cells (**a**) and 4T1 cells (**b**) were treated with FTY720 (5 μM) for the indicated times. Histone acetylations in nuclear extracts were detected by immunoblotting with antibodies to specific histone acetylation sites. (**c**) Purified nuclei from naive MCF7 cells were incubated for the indicated times with vehicle, S1P (1 μM) or FTY720-P (1 μM) and histone acetylations determined. (**d**, **e**) Purified nuclei were isolated from MCF7 cells transfected with vector, SphK2 or ciSphK2 and treated with FTY720 (1 μM) for 15 min. (**f**, **g**) Purified nuclei were isolated from MCF7 cells transfected with siControl or siSphK2 and incubated with the indicated concentrations of FTY720 for 15 min. Histone acetylations were determined by immunoblotting (**d**, **f**) and levels of FTY720-P by liquid chromatography, electrospray ionization/tandem mass spectrometry (LC-ESI-MS/MS) (**e**, **g**). *P<0.05. (**h, i**) Naive MCF7 cells were treated with vehicle, FTY720-P (100 nM), FTY720 (1 μM) or SAHA (2 μM) for 2 h, nuclear extracts were analyzed by western blotting with the indicated antibodies (**h**) and HDAC activity measured and expressed as arbitrary fluorescence units (AFU) (**i**). *P<0.001.

Because the majority of FTY720-P is produced in the nucleus of breast cancer cells where its target HDACs are located, we next examined its effects on histone acetylation. Concomitant with increased nuclear FTY720-P (Figure 1), FTY720 increased acetylation of specific lysines of histone H3, H4 and H2B in MCF7 and 4T1 cells (Figures 2a and b). Similar results were found with MDA-MB-231 cells (Supplementary Figure S2a). In order to demonstrate that these effects are because of the intranuclear action of FTY720-P, experiments were carried out with purified MCF7 nuclei devoid of S1PRs. FTY720-P more potently than S1P enhanced histone acetylation in these nuclei (Figure 2c). Treatment of MCF7 cells with the SphK2 inhibitor K145 reduced FTY720-mediated histone acetylation (Supplementary Figure S2b). Importantly, addition of FTY720 itself also increased histone acetylation in these nuclei in a SphK2-dependent manner, as this effect was further enhanced by overexpression of SphK2 (but not catalytically inactive SphK2^G212E^) (Figure 2d) and was prevented by its downregulation (Figure 2f). These effects correlated with the extent of formation of FTY720-P (Figures 2e and g).

To conclusively demonstrate that the effects on histone acetylation are due to direct intranuclear action of FTY720-P on HDAC activity independently of canonical signaling through S1PRs, cells were treated with FTY720-P that activates all S1PRs except S1PR2.^20^ Although treatment of cells with FTY720-P, as expected, increased S1PR-mediated phosphorylation of ERK1/2, it did not induce significant changes in histone acetylation or in HDAC activity, in contrast to the significant effects of treatment with FTY720 or SAHA (Figures 2h and i).

**Figure 3:**
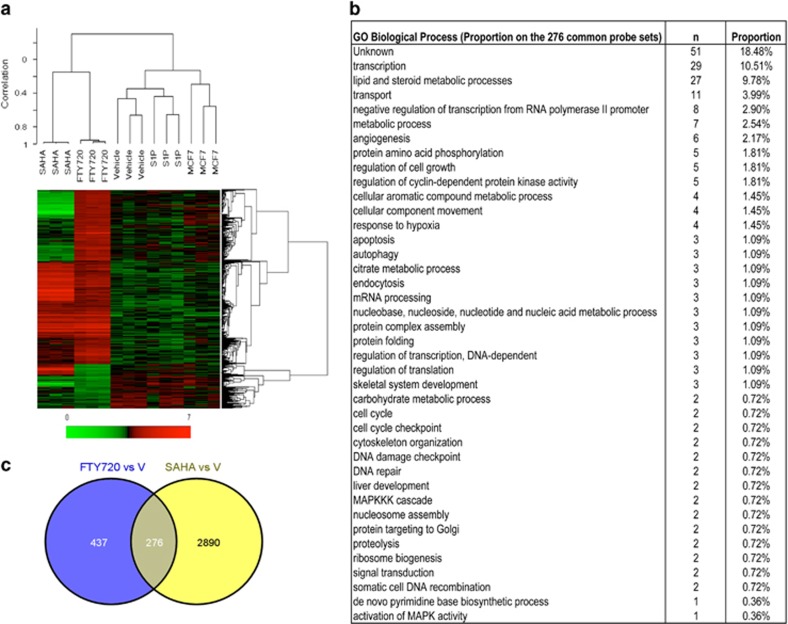
Microarray analysis of genes regulated by FTY720 and SAHA. Gene expression in naive MCF7 cells or MCF7 cells treated with vehicle, S1P (100 nM), FTY720 (1 μM) or SAHA (1 μM) for 24 h was determined by microarray analyses. (**a**) Heatmap showing supervised hierarchical clustering of 713 genes differentially expressed in FTY720-treated cells compared with naive. Expression level of a given gene is indicated by red (high) and green (low). Note that not all of the genes differentially regulated by SAHA are shown. (**b**) Venn diagram of genes differentially regulated by FTY720 and SAHA. (**c**) The gene ontology (GO) Biological Process analyses of 276 common probe sets regulated by SAHA and FTY720 treatment ranked for biological processes.

Microarray analysis also indicated that the effects of FTY720 on gene expression in breast cancer cells are clearly distinguished from S1P receptor occupancy (Figure 3a). Unsupervised cluster analysis on 22 277 probe sets as well as supervised hierarchical cluster analyses demonstrated that there were no major differences in the clustering of the gene expression profiles between the naive, vehicle-treated and S1P-treated groups, whereas significant differences were observed in gene clustering between them and the FTY720- and SAHA-treated groups (Figure 3a). Although activation of S1PRs did not significantly alter gene expression, FTY720 treatment significantly changed the profiles of 713 genes. In comparison, SAHA significantly altered expression of 3166 genes, of which 276 were common to FTY720 (Figure 3b). The gene ontology analysis revealed that the majority of the commonly affected genes were related to transcription followed by lipid and steroid biosynthesis, transport and metabolic processes (Figure 3c). In addition, regulation of cell growth and angiogenesis genes was also prominent. Together, these results indicate that breast cancer cells take up FTY720 and that FTY720-P produced in the nucleus by SphK2 inhibits class I HDACs and increases specific histone acetylations and regulates expression of a restricted subset of gene programs independently of S1PRs.

### FTY720 treatment suppresses development and progression of spontaneous breast tumors in HFD-fed MMTV-PyMT transgenic mice

**Figure 4:**
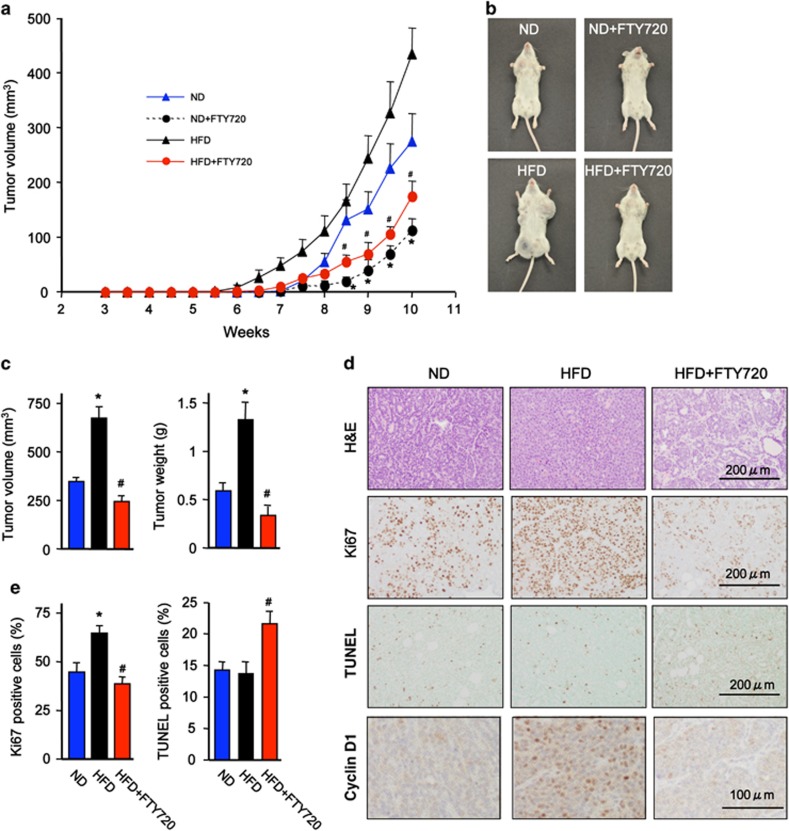
FTY720 treatment suppresses advanced tumorigenic mammary lesions in HFD-fed PyMT transgenic mice. Female PyMT transgenic mice were fed with a normal diet (ND) or a Western HFD, and were treated daily with saline or FTY720 (1 mg/kg) by gavage starting after weaning. (**a**) Tumor volumes were determined at the indicated times. (**b**) Representative images of 10-week-old female PyMTTg mice fed with ND or HFD without or with FTY720. Note the difference in the size of the tumors in the mammary pads. (**c**) Tumor volumes and weights were determined at 11 weeks. (**d**, **e**) Tumor sections were stained with hematoxylin and eosin (H&E), proliferation determined by Ki67 staining, apoptosis by TUNEL and cyclin D1 expression determined by immunohistochemistry. Scale bars: 200 μm and 100 μm, as indicated. (**e**) Quantification of Ki67- and TUNEL-positive cells. Data are mean±s.e.m. ^#^*P*<0.05 compared with ND; *P<0.05 compared with HFD.

Aberrant expression of class I HDACs and dysregulation of global histone acetylations has been found in a many cancers, including breast, and HDACs are promising targets in cancer therapeutics (reviewed in refs 21, 22, 23, 24). Therefore, it was of interest to examine whether the HDAC inhibitory activity of FTY720-P could mitigate breast cancer development in a mouse model. Because diet, particularly fat intake, contributes to the development and progression of breast cancer and has been associated with worse prognosis,^4, 5, 25^ MMTV-PyMT transgenic mice, which spontaneously develop breast cancer that closely mimics progression of the human disease,^26, 27^ were fed a high-fat diet (HFD). Female PyMT transgenic mice on a normal chow diet spontaneously developed palpable mammary tumors by 7.5 weeks (Figure 4a). In agreement with others,^28, 29^ feeding a HFD accelerated the onset of tumors that were palpable by 6 weeks (Figure 4a) and increased tumor multiplicity and size (Figures 4a and b). Although FTY720 administration decreased tumor burden without affecting onset in mice on normal diet (Figure 4a), it significantly increased the latency for appearance of palpable tumors to 7.5 weeks and dramatically suppressed tumor development in mice on HFD (Figures 4a–c). In addition, HFD-fed PyMT mice exhibited more advanced mammary carcinogenic lesions with poorly differentiated malignant cells of dissimilar cell shape and size (Figure 4d). These changes were all mitigated by FTY720 treatment. Consistent with the profound effect on tumor size, there was a significant increase in proliferation determined by Ki67 staining in the mice fed HFD compared with normal diet, which was decreased by FTY720 (Figures 4d and e). Conversely, TUNEL (terminal deoxynucleotidyl transferase dUTP nick end labeling) staining revealed a large increase in apoptotic cells in tumors from FTY720-treated MMTV-PyMT transgenic mice (Figures 4d and e).

### FTY720 treatment reverses HFD-induced HDAC activity and loss of estrogen and progesterone receptors in advanced carcinoma

**Figure 5:**
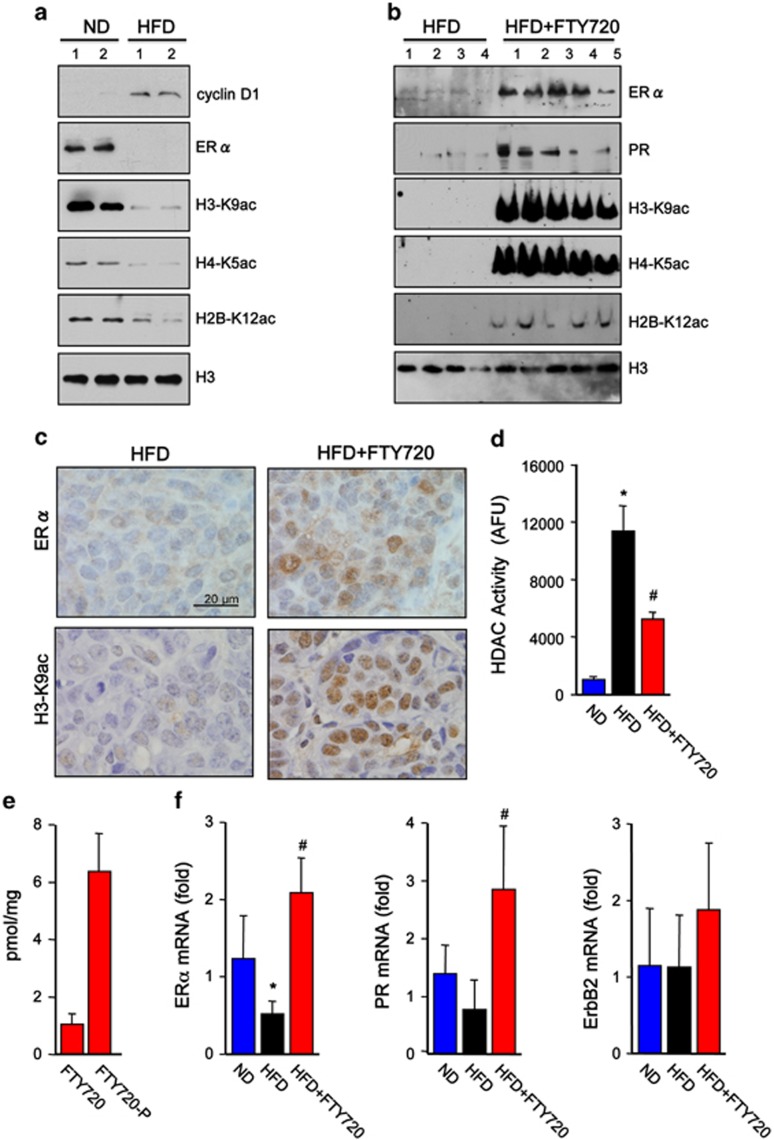
FTY720 treatment reverses HFD-induced loss of estrogen and progesterone receptors in PyMT transgenic mice. Female PyMT transgenic mice were fed with a normal diet (ND) or a Western HFD, and were treated daily with saline or FTY720 (1 mg/kg) by gavage starting after weaning (*n*=5 each), as indicated. (**a**, **b**) Nuclear extracts from tumors were analyzed by western blotting with the indicated antibodies. (**c**) Representative images of tumor sections immunostained with anti-ERα or anti-H3-K9ac antibodies. Scale bars: 20 μm. (**d**) HDAC activity in nuclear extracts of tumors was determined and expressed as arbitrary fluorescence units. (**e**) FTY720 and FTY720-P levels in nuclear extracts of tumors from mice on HFD treated with FTY720 were measured by liquid chromatography, electrospray ionization/tandem mass spectrometry (LC-ESI-MS/MS). (**f**) ERα, PR and ErbB2 mRNA levels in tumors were quantified by quantitative real-time PCR (QPCR) and normalized to *Gapdh*. Data are mean±s.e.m. *P<0.05 compared with ND; ^#^*P*<0.05 compared with HFD.

In agreement with the advanced carcinoma observed in animals fed with HFD and consistent with a previous report,^29^ expression of cyclin D1 was elevated (Figures 4d and 5a) and immunohistochemistry revealed high intensity of cyclin D1-positive clusters within these tumors (Figure 4d). In contrast, ERα protein and mRNA as well as PR mRNA were significantly reduced (Figure 5a and f), all of which are associated with poor prognosis in human breast cancers.^30^ Notably, these characteristics of advanced tumorigenic mammary lesions were reversed by FTY720 administration to the HFD-fed transgenic mice (Figure 5b). Moreover, FTY720 treatment clearly induced nuclear ERα expression (Figure 5b, c, and f). Intriguingly, HFD reduced acetylation of H3-K9, H4-K5 and H2B-K12 in breast tumors that corresponded with increased nuclear HDAC activity (Figures 5a). Conversely, FTY720 administration dramatically increased these specific histone acetylations determined by immunoblotting (Figure 5b) and confirmed by immunohistochemical staining of H3-K9ac in mammary tumors (Figure 5c). FTY720 also reduced HDAC activity in breast tumors (Figure 5d), concomitant with marked production of nuclear FTY720-P compared with FTY720 (Figure 5e) and increased mRNA levels of ERα and PR, without affecting expression of ErbB2 (Figure 5f). Interestingly, in tumor-free mammary fat pads from MMTV-PyMT transgenic mice or from naive C57BL/6 mice, HFD reduced HDAC activity and increased histone acetylations (Supplementary Figure S3).

### FTY720 sensitizes triple-negative breast cancer cells to TAM by reactivation of silenced ERα expression

**Figure 6:**
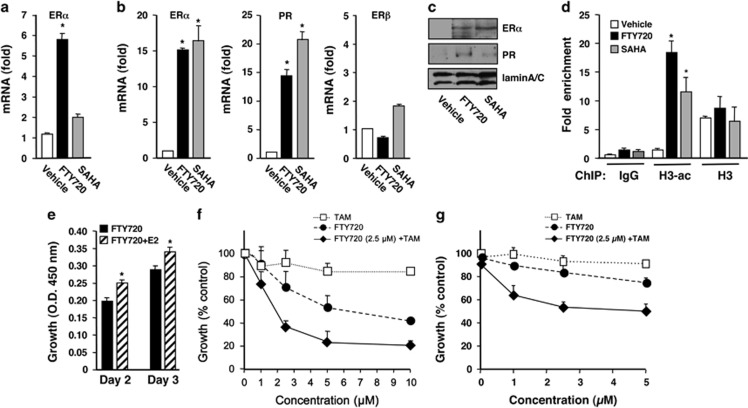
FTY720 induces ERα expression in ERα-negative human and murine breast cancer cells and sensitizes them to tamoxifen. 4T1 (**a**) and MDA-MB-231 (**b**) cells were treated with FTY720 (5 μM) or SAHA (1 μM) for 24 h. ERα, PR and ERβ mRNA levels were determined by quantitative real-time PCR (QPCR) and normalized to GAPDH. (**c**) Proteins in MDA-MB-231 nuclear extracts were analyzed by immunoblotting with the indicated antibodies. LaminA/C was used as a loading control. (**d**) MDA-MB-231 cells were subjected to chromatin immunoprecipitation (ChIP) analyses with antibodies to H3-ac, H3 or normal rabbit IgG, as indicated. The precipitated DNA was analyzed by real-time PCR with primers amplifying the core promoter sequence of the ERα gene. Relative binding to the promoter is expressed as fold enrichment compared with input. Data are mean±s.d. *P<0.003 compared with vehicle. (**e**) MDA-MB-231 cells were treated with FTY720 (1 nM) without or with 10 nM E2 for the indicated days and cell proliferation was determined by WST assay. (**f**, **g**) MDA-MB-231 cells (**f**) or 4T1 cells (**g**) were treated with the indicated concentrations of TAM or FTY720, or with 2.5 μM FTY720 with increasing concentrations of TAM for 48 h and cell proliferation determined. Data are expressed as % of untreated control.

HDACs are negative regulators of the ERα transcriptional complex and HDAC inhibitors have been shown to epigenetically restore ERα expression and reverse TAM resistance in hormone-resistant breast cancer cells^6, 7, 31, 32^ and in preclinical animal studies.^33, 34^ As we found that FTY720 is phosphorylated in the nucleus of ERα-negative MDA-MB-231 and 4T1 breast cancer cells by SphK2 (Figure 1) and that FTY720-P is a potent class I HDAC inhibitor, we asked whether it induces ERα re-expression in ERα-negative breast cancer. Although FTY720 alters expression of many fewer genes than SAHA (Figure 3c), FTY720 more potently than SAHA enhanced ERα expression in 4T1 cells (Figure 6a), whereas in MDA-MB-231 cells, it enhanced ERα mRNA and protein expression to the same extent as SAHA (Figures 6b and c). Similar to SAHA, FTY720 also enhanced expression of PR, one of the ERα target genes (Figures 6b and c). As expected, neither SAHA nor FTY720 had a significant effect on expression of ERβ (Figure 6b). Moreover, chromatin immunoprecipitation assays revealed that FTY720, even more potently than SAHA, enhanced association of acetylated histone H3 at the ERα promoter (Figure 6d), indicating that reactivation of ERα expression correlates with ERα promoter hyperacetylation. In agreement, treatment of these ERα-negative cells with E2 enhanced their proliferation only in the presence of FTY720 (Figure 6e). We next examined whether FTY720, which restores ERα expression in ERα-negative breast cancer cells, could also induce sensitivity to TAM, an ERα antagonist. As expected, TAM alone at concentrations up to 10 μM did not inhibit growth of MDA-MB-231 cells (Figure 6f). Like other HDAC inhibitors,^6, 31, 32^ FTY720 reduced growth in a concentration-dependent manner and importantly sensitized the cells to TAM. For example, although a concentration of 2.5 μM TAM or FTY720 alone only reduced growth of MDA-MB-231 cells by 9.3% or 29.3%, respectively, when combined, cell growth was inhibited by >63% (Figure 6f), with a Synergistic Index of 0.23. Similarly, in highly metastatic ERα-negative 4T1 murine mammary carcinoma cells, FTY720 also greatly enhanced the growth inhibitory effect of TAM (Figure 6g), with a Synergistic Index of 0.23.

### FTY720 increases therapeutic sensitivity of ERα-negative syngeneic breast tumors to TAM

**Figure 7:**
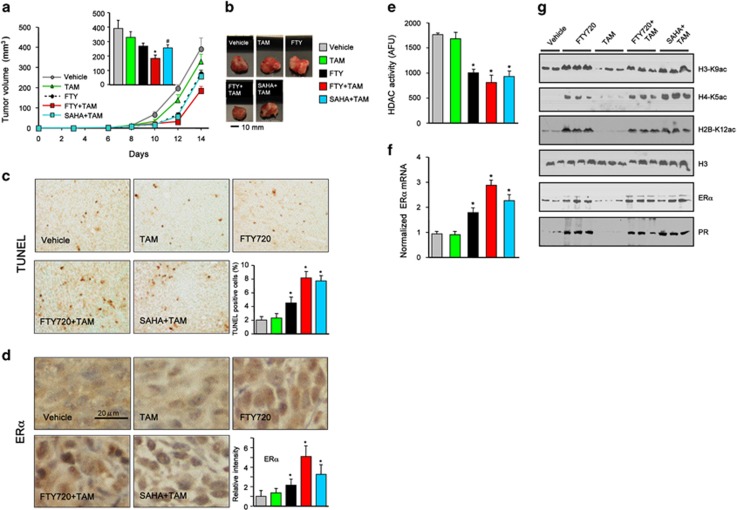
FTY720 reduces breast tumor growth and enhances anticancer effectiveness of TAM in ERα-negative 4T1 syngeneic xenografts. 4T1 cells were surgically implanted into the second mammary fat pads under direct vision. Tumor-bearing mice were randomized into five groups 2 days after implantation and then treated with vehicle, FTY720 (1 mg/kg), TAM (25 mg/kg), FTY720 plus TAM or SAHA (intraperitoneal (i.p.) 20 mg/kg) plus TAM by gavage daily till day 15 (*n*=8). (**a**) Tumor volumes were measured daily. (Insert) Tumor volumes on day 15. (**b**) Representative tumors. *P<0.01, ^#^*P*<0.05 compared with vehicle. (**c**, **d**) Immunohistochemical staining of tumor sections for TUNEL (**c**) and ERα (**d**). Scale bar: 20 μm. Quantifications of TUNEL-positive cells and ERα intensity are shown. *P<0.05 compared with vehicle or TAM. (**e**) HDAC activity in nuclear extracts of tumors was determined and expressed as arbitrary fluorescence units. (**f**) Expression of ERα in the tumors was analyzed by quantitative real-time PCR (QPCR) and normalized to *Gapdh*. (**g**) Nuclear extract proteins were analyzed by western blotting with the indicated antibodies. Histone H3 was used as loading control. Data are mean±s.e.m. *P<0.01 compared with vehicle or TAM.

As we have found that FTY720 treatment induces functional ERα reactivation and sensitizes ERα-negative breast cancer cells to TAM *in vitro*, we sought to determine whether FTY720 also enhances antiestrogen therapy *in vivo*. We utilized a syngeneic mouse metastatic breast cancer model instead of conventional xenografts in immunocompromised nude mice that more accurately mimics human breast cancer.^35, 36^ ERα-negative 4T1 cells were orthotopically implanted into the second mammary fat pad of immunocompetent mice and randomized to insure similar tumor burdens before treatment. 4T1 cells produced large primary tumors in the chest mammary fat pad that were not significantly reduced by TAM administration (Figure 7a). Orally administered FTY720 reduced tumor growth, an effect that was significantly potentiated by coadministration of TAM (Figures 7a and b). Strikingly, FTY720 enhanced the antitumor efficacy of TAM more than SAHA. TUNEL staining also revealed a large increase in apoptotic cells in tumors from FTY720- plus TAM-treated mice as compared with tumors from mice treated with each separately (Figure 7c). Immunohistochemical analysis revealed that nuclear expression of ERα was increased in tumors from FTY720-treated mice that was more prominent when combined with TAM than even in tumors from mice treated with the combination of SAHA and TAM (Figure 7d). Consistent with the decreased nuclear HDAC activity in tumors from animals treated with FTY720 or SAHA (Figure 7e), acetylation of histone H3-K9, H4-K5 and H2B-K12 was increased in these tumors (Figure 7g), leading to re-expression of ERα (Figures 7f and g). Taken together, these data suggest that FTY720 induces epigenetic ERα reactivation *in vivo* to enhance hormonal therapy of ERα-negative breast cancer.

## Discussion

Hormonal therapies, including selective estrogen receptor modulators and aromatase inhibitors, are the standards of care for treatment of ER-positive breast cancer. However, development of resistance to hormone therapies in advanced breast cancer is a major obstacle. Therefore, epigenetic reactivation of silenced ERα expression by HDAC inhibitors has emerged as an attractive potential mode of therapy for these breast cancer patients.^9^ Moreover, treatment of triple-negative breast cancer, which has poor prognosis, remains challenging because the tumors are more aggressive and resistant to hormonal therapy.^30^ Epigenetic modifications are responsible for the lack of ERα expression and HDACs 1, 2 and 3 are overexpressed in breast cancer and correlate with more aggressive tumor type.^37^ In agreement, we found that consumption of HFD by MMTV-PyMT transgenic mice induced more aggressive, poorly differentiated tumors with increased HDAC activity. In contrast, in mammary pads from naive animals or those without tumors, HFD reduced HDAC activity, supporting the notion that HFD and obesity increase acetylations of histones with changes in the epigenome.^38^ Similar to our findings, it was previously reported that HFD-induced HDAC activity plays important roles in epigenetic regulation of tumor suppressor genes involved in colorectal tumor growth and progression, including increased epithelial–mesenchymal transition and tumor inflammation in a xenograft model.^39^

Several HDAC inhibitors have been developed that restored the efficacy of hormonal therapy in preclinical models^33, 34, 40^ and a few have advanced to clinical trials.^9, 41^ Combination of the HDAC inhibitor vorinostat with TAM for patients with ER-positive metastatic breast cancer progressing on hormonal therapy showed encouraging reversal of hormone therapy resistance.^9^ Similar results were obtained in a phase II clinical trial in postmenopausal women with locally recurrent or metastatic ER-positive breast cancer progressing on treatment with a nonsteroidal aromatase inhibitor when combined with the HDAC inhibitor entinostat,^41^ leading to a phase III clinical trial that is currently underway (Clinical-Trials.gov identifier: NCT02115282).

It was originally suggested that the prodrug FTY720 (fingolimod, Gilenya) approved for human use is phosphorylated at the plasma membrane by SphK2 to form FTY720-phosphate that acts via S1P receptors.^10, 20^ However, in this study we have demonstrated that FTY720 is predominantly phosphorylated to FTY720-P in the nucleus of both ER-positive and ER-negative breast cancer cells. Although it has been suggested that phosphorylation of FTY720 by SphK2 is a requirement for its induction of apoptosis *in vitro* and *in vivo*, the targets for this action have not been identified.^42^ We are now showing that the active phosphorylated form of FTY720 is a potent class I HDAC inhibitor. This novel nuclear action of FTY720-P provides a new mechanism to explain the cytotoxic effects of FTY720 in cell culture and its preclinical antitumor efficacy in many xenograft and syngeneic cancer models.^43^ Moreover, similar to other HDAC inhibitors, treatment with FTY720 enhances histone acetylation at the ERα promoter leading to its re-expression, and sensitizes ER-negative breast cancer cells to TAM therapy. Interestingly, even in HFD-fed PyMT transgenic mice that developed more advanced, poorly differentiated mammary tumors with increased HDAC activity and decreased expression of ERα and PR, oral administration of FTY720 not only suppressed development and progression of these spontaneous breast tumors, but also reduced HDAC activity in tumors and concomitantly induced expression of ERα and PR. Importantly, FTY720 treatment of breast tumor-bearing mice also induced re-expression of ERα in the tumor and greatly enhanced the anticancer efficacy of TAM, even more potently than a known HDAC inhibitor.

FTY720 has multiple beneficial anticancer activities. First, it has been convincingly shown that the unphosphorylated form is a potent activator of protein phosphatase 2A, a heterotrimeric serine/threonine phosphatase that counteracts the activity of many kinase-driven signaling pathways, including MEK and AKT.^17, 18^ In this regard, reduced protein phosphatase 2A activity is a common event in breast cancer that could predict sensitivity to FTY720.^44^ Second, unphosphorylated FTY720 also inhibits and induces proteasomal degradation of SphK1,^45^ which is upregulated in breast cancer and correlates with poor prognosis and drug resistance.^46, 47, 48, 49^ High expression of SphK1 and S1PR1 are also associated with development of TAM resistance in ER-positive breast cancer patients.^50^ Third, because FTY720-P is a functional antagonist of S1PR1, it can also suppress tumor growth by several S1PR1-dependent mechanisms. It was shown to decrease prosurvival/anti-apoptotic signaling from S1PR1 via suppression of proapoptotic Bim and upregulation of prosurvival Mcl-1 proteins.^51^ Targeting S1PR1 with FTY720 also interferes with a major positive feedback loop for persistent STAT3 activation in breast tumor microenvironment critical for malignant progression.^14^ Moreover, FTY720 by interfering with the upregulation of SphK1 and S1PR1 curtails the S1P/SphK1/S1PR1 feed-forward amplification loop that leads to nuclear factor-κB and persistent STAT3 activation that play important roles in the link between chronic inflammation and cancer.^16^ Finally, in this paper we have uncovered a novel action of FTY720-P as a potent inhibitor of class I HDACs that acts similarly to other HDAC inhibitors to reactivate ERα expression and sensitize breast cancer cells to TAM therapy.

At first glance, the immunosuppressive action of FTY720 would seem to be an undesirable effect in cancer therapy. However, it has been shown that following treatment of tumor-bearing mice with FTY720, there was a significant reduction in accumulation of tumor-associated regulatory T cells and an increase in peripheral blood regulatory T cells, suggesting that FTY720 causes a block in blood-to-tumor regulatory T-cell recruitment that would allow more potent antitumor immunity.^52^ Moreover, treatment of mice with FTY720 after tumors were established to block new T-cell trafficking from secondary lymphoid organs still enabled the increase in the capacity of tumor-infiltrating CD8^+^ T cells to produce IL-2 and to proliferate and subsequent tumor rejection induced by combinatorial immunotherapy with anti-CTLA-4 and anti-PD-L1 monoclonal antibodies.^53^

As HDAC inhibitors are being developed for treatment of breast cancer acting through multiple epigenetic pathways, and numerous clinical trials are underway,^21, 22, 23, 24^ it is not surprising that FTY720 has such potent anticancer activity. FTY720 has several advantages over available HDAC inhibitors as potential treatments for breast cancer patients: it is an orally bioavailable prodrug; it has already been approved for human use; it regulates expression of only a limited number of genes (a majority related to cholesterol and sphingolipid metabolism) compared with other HDAC inhibitors; it has good pharmacokinetics and a long half-life; it suppresses several survival and proliferative pathways; it is much less toxic, accumulates in tumor tissues, and both the phosphorylated and unphosphorylated forms target important pathways in breast cancer. Hence, we hope that our studies will pave the way for exploration of new clinical trials using FTY720 as a prototype of new adjuvant treatment strategies for hormonal-resistant breast cancer.

## Materials and Methods

### Cell culture and transfection

Human breast cancer cells MCF7 and MDA-MB-231 (ATCC, Manassas, VA, USA) and murine 4T1 breast cancer cells (Caliper Life Sciences, Waltham, MA, USA) were cultured and transfected with vector, SphK2 or catalytically inactive SphK2^G212E^ as previously described.^54^ SphK2 was downregulated by transfection with ON-TARGETplus SMARTpool siRNA against SphK2, and scrambled siRNA (Dharmacon, Lafayette, CO, USA) was used as control.^54^ Cell growth was determined with WST-8 reagent and absorbance was measured at 450 nm.^55^

### Nuclear extracts and immunoblotting

Nuclear extracts from tissues and cells were prepared and protein expression determined by immunoblotting as previously described.^54^ Proteins were separated by SDS–polyacrylamide gel electrophoresis, transblotted to nitrocellulose and incubated with primary antibodies as indicated in figure legends, including rabbit polyclonal antibodies to: histone H3-K23ac (1:1000 dilution; EMD Millipore, Billerica, MA, USA); histone H3, H3-K9ac, H4-K5ac and H2B-K12ac (1:1000 dilution; Abcam, Cambridge, MA, USA); laminA/C, tubulin, p-ERK1/2, HDAC3 and HDAC7 (1:1000 dilution; Cell Signaling Technology, Danvers, MA, USA); HDAC1, HDAC2 and HDAC8 (1:1000 dilution; Santa Cruz Biotechnology, Santa Cruz, CA, USA); V5 (1:5000 dilution; Life Technologies, Grand Island, NY, USA); and SphK2 (1:1000 dilution).^54^ Immunopositive bands were visualized by enhanced chemiluminescence using secondary antibodies conjugated with horseradish peroxidase (goat anti-rabbit or anti-mouse, 1:5000 dilution; Jackson ImmunoResearch, West Grove, PA, USA) and Super-Signal West Pico chemiluminescent substrate (Pierce Chemical Co., Rockford, IL, USA). Optical densities of bands associated with proteins of interest were quantified using AlphaEaseFC software (Alpha Innotech, Miami, FL, USA) and normalized to the optical densities of their respective tubulin bands.

### Quantification of sphingolipids by mass spectrometry

Sphingolipids were measured by liquid chromatography, electrospray ionization–tandem mass spectrometry (4000 QTRAP, AB Sciex, Framingham, MA, USA).^54^

### HDAC activity measurements

Enzymatic activities of HDACs immunoprecipitated from nuclear extracts with specific antibodies were measured with fluorometric assay as previously described.^54^.

### Quantitative real-time PCR

Total RNA from cells or tumors was isolated with Trizol (life Technologies, Grand Island, NY, USA) and reverse transcribed with the High Capacity cDNA Reverse Transcription Kit and pre-mixed primer probe sets from Applied Biosystems (Foster City, CA, USA). Complementary DNA (cDNA) was amplified with the ABI 7900HT (Applied Biosystems).^54^

### Chromatin immunoprecipitation

Cells were crosslinked with 1% formaldehyde for 10 min at 37 °C and then quenched with glycine, washed with cold phosphate-buffered saline, suspended in SDS buffer, sonicated and centrifuged. Supernatants were pre-cleared with protein G-Sepharose beads (GE Healthcare, Piscataway, NJ, USA) that were blocked with sonicated herring sperm DNA (Promega, San Louis Obispo, CA, USA) in IP buffer (16.7 mM Tris (pH 8), 16.7 mM NaCl, 1.2 mM EDTA, 0.01% SDS, 1.1% Triton X-100, containing 0.05 mg/ml bovine serum albumin). Chromatin was immunoprecipitated with rabbit polyclonal anti-acetylated H3 or anti-H3 antibodies, or with control rabbit IgG.^54^ DNA–protein complexes were pulled down with the protein G-Sepharose beads and then washed with low salt buffer, high salt buffer, LiCl buffer and Tris-EDTA buffer before eluting with 1% SDS in 0.1 M NaHCO_3_. Crosslinks were reversed by heating at 65 °C overnight in 0.3 M NaCl, followed by proteinase K digestion for 1 h at 55 °C. Input samples were also similarly treated. DNA was purified with QIAquick PCR Purification Kit (Qiagen, Germantown, MD, USA). The ER promoter was analyzed by quantitative real-time PCR using SYBR Green Master Mix (Applied Biosystems) and the following primers: sense, 5′-GAACCGTCCGCAGCTCAAGATC-3′ anti-sense, 5′-GTCTGACCGTAGACCTGCGCGTTG-3′. Results were analyzed relative to input using the ΔCT method. Specific endogenous chromatin immunoprecipitation enrichments were all at least threefold greater than control.

### Animal studies

All animal studies were conducted in the Animal Research Core Facility at VCU School of Medicine in accordance with the institutional guidelines. Animals were bred and maintained in a pathogen-free environment and all procedures were approved by the VCU Institutional Animal Care and Use Committee that is accredited by the Association for Assessment and Accreditation of laboratory Animal Care. All mice were kept on a 12-h light/dark cycle with free access to food.

Male MMTV-PyMT mice on a FVB/N background (Jackson Laboratories, Bar Harbor, MD, USA) were randomly bred with normal FVB/N females to obtain females heterozygous for the PyMT oncogene. Mice were fed a normal diet or HFD (TD.88137; Harlan Laboratories, Indianapolis, IN, USA) containing cholesterol (0.2%), total fat (21% by weight; 42% kcal from fat), saturated fatty acids (>60% of total fatty acids), sucrose (34% by weight), protein (17.3% by weight) and carbohydrate (48.5% by weight). Palpable mammary tumors developed as early as 6 weeks of age. Tumor size was measured with calipers every 3 days and total tumor volume was estimated by the cylinder formula.

For the syngeneic breast cancer model, 4T1 mouse mammary cancer cells were surgically implanted in the upper fat pads of female BALB/c mice (8 to 12 weeks of age, Jackson Laboratories) under direct vision as described previously.^35, 36^ Tumor-bearing mice were randomized 2 days after implantation into five treatment groups: vehicle, FTY720 (p.o. 1 mg/kg, Cayman Chemical Company, Ann Arbor, MI, USA), TAM (intraperitoneal 25 mg/kg; Sigma-Aldrich, St Louis, MO, USA), FTY720 plus TAM, and SAHA (intraperitoneal 20 mg/kg; Sigma-Aldrich) plus TAM. Tumors were measured regularly and tumor volumes calculated. At the indicated times, animals were killed by exsanguination, blood was collected, tumors excised, weighed, fixed in 10% neutral buffered formalin and embedded in paraffin or frozen in liquid nitrogen for morphological and immunofluorescence analyses.

### Histopathological analysis

Tissue slices (5 μm) were stained with hematoxylin and eosin for morphological analysis. Frozen tissue samples were embedded in Optimal Cutting Medium (OCT 4583; Sakura Finetek, Torrance, CA, USA) for immunofluorescence analysis. Paraffin-embedded slides were deparaffinized, and antigen unmasking was carried out by microwave heating in citrate buffer for 20 min. Slides were incubated with 3% H_2_O_2_ and then with goat or horse serum (DAKO, Carpinteria, CA, USA) for 30 min at room temperature. After washing with phosphate-buffered saline, slides were incubated at 4 °C overnight with the following primary antibodies: ERα (Santa Cruz), H3-K9ac (Abcam), cyclin D1 (Cell Signaling) and Ki67 (Dako). Biotinylated secondary antibodies were added and incubated at room temperature for 20 min. After 5 min with streptavidin-HRP, sections were stained with DAB substrate and counterstained with hematoxylin. Slides were examined with a Zeiss Axioimager A1 (Jena, Germany) and images captured with an AxioCam MRc camera.

### Gene expression microarrays

Total RNA was extracted using the MagMAX-96 for Microarrays Total RNA Isolation Kit (Life Technologies) in an automated manner using the MagMAX Express magnetic particle processor. RNA purity integrity was assessed by spectrophotometry at 260, 270 and 280 nm and by RNA 6000 Nano LabChips with the 2100 Bioanalyzer (Agilent Technologies, Carpenteria, CA, USA).^56^ Single-strand cDNA was synthesized from 500 ng total RNA primed with a T7-(dT24) oligonucleotide. Second-strand cDNA synthesis was performed with *Escherichia coli* DNA Polymerase I, and cRNA biotinylated by *in vitro* transcription using the GeneChip 3' *in vitro* transcription Express Kit (Affymetrix, Santa Clara, CA, USA). After incubation at 37 °C for 16 h, labeled cRNA was purified using the GeneChip Sample Cleanup Module. Fragmented cRNA (10 μg) was hybridized on the GeneChip HG-U133A 2.0 array for 16 h at 60 r.p.m. in a 45 °C hybridization oven. Arrays were washed and stained with streptavidin phycoerythrin (Life Technologies) in the Affymetrix fluidics workstation. Every chip was scanned at a high resolution on the Affymetrix GeneChip Scanner 3000 7G and raw intensities for every probe were stored in electronic files by the GeneChip Operating Software v1.4.^36^ Overall quality of each array was assessed by monitoring the 3′/5′ ratios for the housekeeping gene, *glyceraldehyde 3-phosphate dehydrogenase* (*GAPDH*), and the percentage of ‘Present' genes (%P). Arrays exhibiting *GAPDH* 3′/5′ <3.0 and %*P* >40% were considered good-quality arrays. For microarray data analyses, background correction, normalization and estimation of probe set expression summaries and filtering and hierarchical cluster analyses were performed using the log-scale Robust Multi-array Analysis method^57^ and BRB-ArrayTools v3.1.0 (NCI, Bethesda, MD, USA), respectively. Differentially expressed genes among the classes were identified by *t*-test analyses. To adjust for multiple hypotheses testing, the resulting *P*-values were used to obtain the false discovery rates using the *q-*value method. All analyses were performed on the R environment using functions provided by the BioConductor packages.^58^

### Statistical analysis

Statistical analysis was performed using unpaired two-tailed Student's *t-*test for comparison of two groups. *P*<0.05 was considered significant. Experiments were repeated at least three times with consistent results. For animal studies, measurements were blind with respect to group assignments.
