# Supplementary material for: The phosphorylated prodrug FTY720 is a histone deacetylase inhibitor that reactivates ERα expression and enhances hormonal therapy for breast cancer
Source: Oncogenesis. 2015 Jun 8;4(6):e156–. doi: 10.1038/oncsis.2015.16 (PMC4753524; doi:10.1038/oncsis.2015.16)
Supplement: Supplementary Information [file oncsis201516x1.docx]

**Supplementary Data**


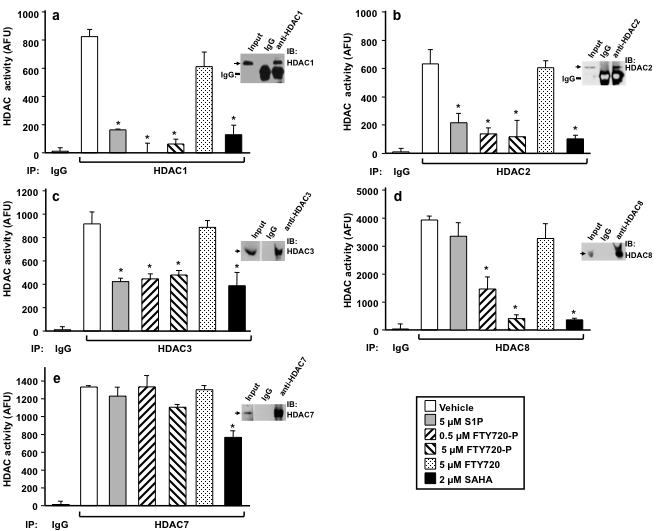


**Supplementary Figure S1. FTY720-P inhibits activity of endogenous class I HDACs.** Nuclear extracts of HeLa cells were immunoprecipitated with control IgG or the indicated HDAC-specific antibodies. Immunoprecipitates were washed, and HDAC activities were measured in the presence of vehicle, S1P (5 μM), FTY720-P (0.5 or 5 μM), FTY720 (5 µM), or SAHA (2 μM). Data are averages of triplicate determinations ± SD and expressed as arbitrary fluorescence units (AFU). *, P < 0.01, compared to Vehicle. Insets: Immunoprecipitates were analyzed by western blotting with HDAC-specific antibodies.


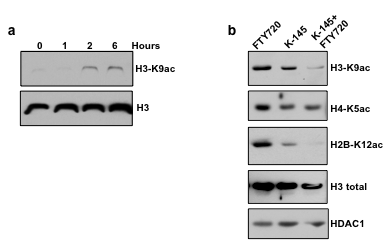


**Supplementary Figure S2. FTY720 is phosphorylated by nuclear SphK2 in MDA-MB-231 and MCF7 human breast cancer cells. (a)** MDA-MB-231 cells were treated with FTY720 (5 μM) for the indicated times. **(b)** MCF7 cells were pretreated with 1 µM K-145 for 30 min and then 1 µM FTY720 was added and the cells were incubated for an additional 4 h. Histone acetylations in nuclear extracts were detected by immunoblotting with the indicated antibodies.


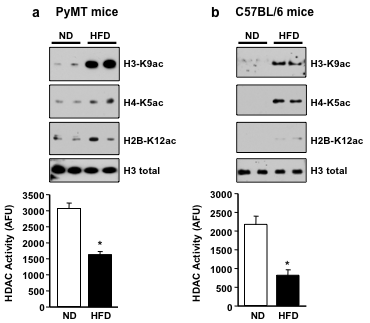


**Supplementary Figure S3. HFD consumption reduces HDAC activity and increases histone acetylation in tumor-free mouse mammary fat pads.** Female PyMT transgenic mice (**a**) or naïve female C57BL/6 mice (**b**) were fed a normal diet (ND) or a HFD for 14 weeks and 15 months, respectively, and tumor-free mammary fat pads were removed. Nuclear extracts from mammary pads were analyzed by western blotting with the indicated antibodies. HDAC activity in nuclear extracts was determined and expressed as arbitrary fluorescence units. *, P < 0.01, compared to ND.
